# A Manual of Pathological Anatomy

**Published:** 1855-10

**Authors:** 


Art. VII.—*A Manual of Pathological Anatomy.* By Carl Rokitansky, M. D., Curator of the Imperial Pathological Museum, and Professor at the University of Vienna, etc. Translated from the last German edition by Wm. Edward Swaine, M. D., Edward Sieveking, M. D., Charles Hewitt Moore, Geo. E. Day, M. D., F. R. S. Four volumes in two. Philadelphia: Blanchard & Lea. 1855. 8vo., pp. 586.

The long promised work of Dr. Carl Rokistansky is at length within reach of the American practitioner. We at last have a Pathological Anatomy in which things are described as they were actually seen in the dead-house by one who was unfettered by preconceived notions or prejudiced views as to the disease of the individual examined.

"ROKITANSKY," as Mr. WILDE justly remarks, "differs from all other pathologists, in not engaging in the study or treatment of disease during life; he is not a practical physician, and seldom sees one of the many hundreds of cases, whose bodies he dissects."

The present work is based upon an experience running through a long series of years of a man who is represented on all hands to possess acute judgment, untiring industry and a spirit of candid research. There have been made under his eyes more than 30,000 cadaveric sections. Records of every case, taken down at his dictation, are kept, and all interesting specimens are preserved for the Pathological Museum. The facts observed and the conclusions deduced from them are embodied in the beautiful book before us.

"Whilst engaged," he says, "in working out the design of this Pathological Anatomy, I have throughout endeavored to act the part of a clinical teacher; and I believe that, in so doing, I have apprehended the requirements of our day, and usefully disposed of the colossal materials within my reach.

"The same self-reliance that characterised the commencement of my pathologico-anatomical studies, has stood by me whilst engaged in observing and interpreting the facts of which said materials are composed; for, each individual discovery encouraged me more and more to pin my faith upon nature alone. Still I have never failed to watch and to appreciate the achievements of other men.

"The present work will, at any rate, tend to show how thorough is my conviction that Pathological Anatomy must constitute the groundwork, not alone of all medical knowledge, but also of all medical treatment, nay, that it embraces all that medicine has to offer of positive knowledge, or at least of what is fundamental to it. Its domain will here, however, be found more extended, and more nearly approximated to the confines of Pathological Chemistry than has generally been the case in pathologico-anotomical writings."

The translation was executed by four gentlemen, each well qualified for the portion entrusted to him, and was published in London under the auspices of the Sydenham Society—a guarantee of its fidelity.

By presenting this "great store-house of pathological knowledge, in a convenient and accessible form," Messrs Blanchard & Lea have laid the profession in the United States under renewed obligations to them. The work is, in every respect, a first class publication. D. W. Y.

